# Association of periodontitis and diabetic macular edema in various stages of diabetic retinopathy

**DOI:** 10.1007/s00784-021-04028-x

**Published:** 2021-06-22

**Authors:** Marlene Lindner, Behrouz Arefnia, Domagoj Ivastinovic, Harald Sourij, Ewald Lindner, Gernot Wimmer

**Affiliations:** 1 Department of Dental Medicine and Oral Health, Medical University of Graz, Billrothgasse 4, 8010 Graz, Austria; 2 Department of Ophthalmology, Medical University of Graz, Auenbruggerplatz 4, 8036 Graz, Austria; 3 Department of Internal Medicine, Medical University of Graz, Auenbruggerplatz 15, 8036 Graz, Austria

**Keywords:** Periodontitis, Diabetic macular edema, Diabetic retinopathy, Periodontal inflamed surface area, Optical coherence tomography, Clinical attachment level

## Abstract

**Objectives:**

Periodontitis and diabetes are known to have a bidirectional relationship. Diabetic macular edema is a complication of diabetes that is strongly influenced by inflammatory pathways. However, it remains to be established whether inflammation at other locations, such as periodontitis, affects diabetic macular edema. Here, we investigated the prevalence of periodontitis in patients treated for diabetic macular edema.

**Materials and methods:**

Patients with diabetic macular edema were recruited for this cross-sectional study at the Medical University of Graz. Macular edema was documented by optical coherence tomography. Periodontal status was assessed by computerized periodontal probing and panoramic X-ray imaging. Bleeding on probing, clinical attachment level, probing pocket depth, and plaque index were compared between different stages of diabetic retinopathy.

**Results:**

Eighty-three eyes of 45 patients with diabetic macular edema were enrolled. Forty-four eyes (53.0%) had early stages of diabetic retinopathy (mild and moderate), and 39 eyes (47.0%) had late stages (severe and proliferative). Patients with mild or moderate DR were more likely to have more severe periodontal conditions than patients with severe or proliferative DR. Fourteen patients with mild DR (82.4%), 7 patients with moderate DR (87.5%), 4 patients with severe DR (100.0%), and 15 patients with proliferative DR (93.8%) had some degree of PD. The periodontal inflamed surface areas and the percentages of tooth sites that bled on probing were significantly higher in patients with early stages of diabetic retinopathy than in those with late stages of the disease (p < 0.05). Patients with periodontal inflamed surface areas of more than 500 mm^2^ required significantly more intravitreal injections in the last year than those with milder forms of periodontitis (n = 6.9 ± 3.1 versus n = 5.0 ± 3.5, *p* = 0.03).

**Conclusion:**

In patients with diabetic macular edema, periodontitis is more prevalent in early stages of diabetic retinopathy. We suggest regular dental check-ups for diabetic patients, especially when diabetic macular edema is already present.

**Clinical relevance:**

Patients with diabetic macular edema should be screened for periodontitis and vice versa, particularly early in the course of diabetes.

## Introduction

Diabetes mellitus (DM) presents a major health burden. The total number of people with DM is projected to increase to 366 million by 2030 [1]. In 2012, the estimated total economic cost of DM in the USA alone was 245 billion dollars [2]. Diabetic retinopathy (DR) is a sight-threatening complication of DM that is categorized into mild, moderate, severe, and proliferative stages according to the severity of the resulting vascular lesions, as described by the International Classification of DR [3]. Data from the UK Prospective Diabetes Study showed that 6 years after the diagnosis of DM, 22% of patients developed DR, and 29% of patients progressed two stages or more if DR was already present at the time of DM diagnosis [4].

In addition to DR, DM can lead to an increase in the permeability of retinal capillaries and finally breakdown of the blood retinal barrier, ultimately leading to an accumulation of protein and fluid in the macula, a condition known as diabetic macular edema (DME) [5]. DME is the most common reason for vision loss in people with DM. The prevalence of DME and DR varies greatly among populations, but as a rough estimate, approximately one-third of diabetic patients will develop DR, and of these, approximately one-third will demonstrate signs of DME. The pathophysiology of DME is highly complex, and inflammatory pathways have been suggested to play a pivotal role. For example, a significant elevation in the levels of cytokines in the aqueous humor of patients with DME has been documented [6], and a relationship between systemic inflammatory markers and DME has been shown [7].

The treatment of DME has been revolutionized by the introduction of humanized monoclonal anti-vascular endothelial growth factor (VEGF) antibodies [8]. These antibodies are administered via intravitreal injections (IVIs) and usually lead to a significant reduction in the size of the macular edema. As the antibodies are washed out over time, their therapeutic effect begins to fade, and the macular edema returns, requiring repeated IVIs. The number of IVIs injected per year varies considerably, with frequencies of two [9] to 12 [10] IVIs per year having been reported.

Periodontitis (PD), a known complication of DM, is a pathogenic biofilm-induced inflammatory condition affecting the supportive tissues surrounding the teeth capable of causing tooth and bone loss [11]. Indeed, PD and DM affect each other bidirectionally [12]. Notably, treatment of PD by scaling and root planning lowers glycated hemoglobin (HbA1c) levels, with a mean percentage reduction of 0.29% at 3–4 months [13].

Furthermore, some evidence suggests an interplay between PD and DME. PD is associated with an increase of inflammatory markers in serum and even in aqueous humor [14]. Moreover, PD is associated with impaired endothelial function [15–17], which can also be observed in patients with DME. Recently, PD was also shown to be associated with early age-related macular degeneration, suggesting that subtle systemic changes caused by PD may be relevant only for disease development when other pathogenic influences have not become fully apparent [18]. Treatment of PD can not only reduce the systemic inflammatory burden but also lower the risk for diseases associated with endothelial dysfunction [16]. Hence, investigating the relationship between PD and DME should form the basis for periodontal treatment recommendations for patients with DME. Although several studies have focused on the association between DR and PD, the association between PD and DME remains to be investigated.

Although knowledge about the pathophysiology of DME is accumulating, it remains unclear why some patients with DM develop DME whereas others do not. The duration of DM and hyperglycemia seems to play pivotal roles; however, this does not explain why DME can be present at any stage of DR. Moreover, studies of genetic factors have failed to sufficiently explain this discrepancy [19]. DME, as with other forms of macular edema, is strongly driven by inflammatory pathways. Remote inflammation, such as PD, causes an increase in systemic inflammatory marker levels and could impact DME, potentially explaining why DME develops in patients with mild or no DR.

We designed this study to determine whether there are differences in the distribution of periodontitis in different stages of DR in patients treated for DME.

## Materials and methods

### Patient recruitment and information

Patients undergoing routine IVIs for DME treatment at the Department of Ophthalmology of the Medical University of Graz between October 2018 and February 2019 were recruited for this cross-sectional observational study. Patients were included who had established DM (type 1 or type 2), were aged between 18 and 80 years (exclusively), and were Caucasian, originating from the same region in southern Austria. Patients with any other concomitant ocular disease that could cause macular edema and patients with fewer than 12 teeth were excluded from the study. The patients’ general diseases (e.g., arterial hypertension, hyperlipidemia, systemic inflammatory disease, and osteoporosis), duration of diabetes, HbA1c value, glomerular filtration rate, serum creatinine level, and smoking habits were documented. Numbers of IVIs administered in the last year were assessed by retrospective chart review. This study was approved by the institutional review board of the Medical University of Graz (EK 30–482 ex 17/18), and all participants provided written informed consent. All study procedures were in accordance with the Declaration of Helsinki.

### Ophthalmological examination

All study participants underwent routine ophthalmological examinations including measurement of the best corrected visual acuity with a Snellen chart followed by score conversion to the logarithm of the minimal angle resolution (LogMAR) chart and slit lamp biomicroscopy. Fundus examination was performed after pharmacological pupil dilation. Retinopathy was defined by the presence of characteristic features, such as microaneurysms, hemorrhages, hard exudates, and proliferations, according to the international clinical DR severity scale [3], and classified as mild, moderate, severe, or proliferative. DME was evaluated using spectral domain optical coherence tomography (Spectralis®, Heidelberg Engineering, Heidelberg, Germany), which generates cross-sectional images of the retinal layers [20]. Notably, the central subfield thickness (CST) is a valuable parameter for determining the severity of DME and is automatically calculated by the software. The patients were treated with IVIs of either anti-VEGF antibodies (aflibercept (Eylea®) or bevacizumab (Avastin®)) or the steroid dexamethasone (Ozurdex®).

### Dental examination

Dental examinations were performed at the Department of Dental Medicine and Oral Health of the Medical University of Graz. Radiographic bone loss was evaluated on digital panoramic radiographs. We used three measurements to assess the degree of inflammation in patients with periodontitis: probing pocket depth (PPD), clinical attachment level (CAL), and bleeding on probing (BOP). O’Leary Plaque Index (PI) was determined to evaluate patients’ oral hygiene. Probing pocket depth (PPD) was defined as the distance in millimeters from the gingival margin to the bottom of the pocket. The clinical attachment level (CAL) was defined as the distance from the cementoenamel junction (CEJ) to the location of the inserted probe tip [21]. The PPD and CAL were measured on each tooth with a Florida Probe® (Florida Probe Corp., Gainesville, FL, USA). Bleeding on probing (BOP) and the O’Leary Plaque Index (PI) [22] were also documented. All examinations were performed by the same examiner. To assess PD severity, the periodontal inflamed surface area (PISA) was calculated based on the PPD and BOP measurements using a previously published spreadsheet [23]. The stages of PD were defined according to the new classification of periodontitis [24].

### Statistical analyses

IBM SPSS Statistics version 25.0 was used for all statistical analyses. The Chi-square test was used to compare categorical variables, and Student’s t test or ANOVA was used to compare continuous variables. Corrections for multiple testing were performed with the Bonferroni method. For the post hoc test, each group (mild, moderate, severe, and proliferative) was compared with every other group. The adjusted significance level of the post hoc test was 0.0083. The Kolmogorov–Smirnov test was used to assess the normality of the distributions of continuous data. p Values < 0.05 were considered statistically significant.

## Results

### Patient characteristics

A total of 265 patients who were routinely treated for DME via IVIs between October 2018 and February 2019 were asked to participate in the study. A total of 121 patients declined to be enrolled because of limited mobility or lack of interest, and 99 had to be excluded because they had fewer than 12 teeth. Forty-five patients gave informed consent to participate in the study and were assessed for their periodontal status. The enrolled patients were younger than those who had to be excluded (62.61 ±12.30 vs. 71.28 ±11.55 years; p = 0.0004). This difference was not unexpected, as a low number of teeth was one of the exclusion criteria. Participants enrolled in the study were not significantly different in age from those who declined to participate (62.05 ± 10.80 years vs. 65.54 ± 8.97 years, p = 0.06). Seven patients were only affected by DME in one eye, resulting in the inclusion of a total of 83 eyes. Seven patients had type 1 DM, and 38 patients had type 2 DM. One patient with mild DR and one patient with proliferative DR were smokers (approximately 20 cigarettes per day). Two patients with proliferative DR and one patient with severe DR had diabetic nephropathy. None of the patients indicated the presence of systemic inflammatory disease, osteoporosis, or alcoholism.

### Diabetic retinopathy

To assess the stages of diabetic retinopathy, full ophthalmological examination was performed. The characteristics of patients diagnosed with different DR stages are shown in Table 1. Compared with milder forms of DR, patients with severe DR had significantly higher serum creatinine levels and a significantly higher frequency of arterial hypertension (p < 0.05). The ophthalmologic parameters of the patients’ eyes are depicted in Table 2. All the eyes had some degree of DR, with 29 having mild, 15 having moderate, eight having severe, and 31 having proliferative DR. Most eyes received anti-VEGF antibody IVIs (29 eyes received bevacizumab and 44 received aflibercept) for DME treatment, and 10 eyes were treated with steroid IVIs. The efficacy of the DME treatment was evaluated by the reduction in the CST relative to that measured at the first visit. Central subfield thickness was high due to edema, but efficacy of treatment was shown by a reduction compared to first visit.

**Table 1:** Patient characteristics grouped by stage of diabetic retinopathy

Stage of DR (number of patients)	Mild DR (17)	Moderate DR (8)	Severe DR (4)	Proliferative DR (16)	*p* Value
Age, years (mean ±SD)	67 ±9	68 ±10	58 ±8	60 ±11	0.06
Male sex, n (%)	14 (82.4)	6 (75)	4 (100)	11 (68.8)	0.26
Duration of diabetes, years (mean ±SD)	17 ±10	16 ± 10	11 ±10	19 ±12	0.53
HbA1c, %	7.4 ±1.2	7.0 ±0.8	7.2 ±0.0	7.5 ±1.1	0.60
Glomerular filtration rate, ml/min (mean ± SD)	78.7 ±23.7	68.1 ±30.7	44.5 ±43.0	68.6 ±29.2	0.22
Creatinine, mg/dl (mean ± SD)	1.0 ±0.4	1.5 ±1.2	4.3 ±3.7	1.3 ±0.8	0.001
Smoker, n (%)	1 (5.9)	0 (0.0)	0 (0.0)	2 (12.5)	0.68
Arterial hypertension, n (%)	7 (41.2)	6 (75.0)	4 (100.0)	9 (56.3)	0.03
Hyperlipidemia, n (%)	5 (29.4)	1 (12.5)	1 (25.0)	5 (31.3)	0.46

*DR* diabetic retinopathy, *SD* standard deviation, *HbA1c* hemoglobin A1c

**Table 2:** Patient ophthalmologic parameters grouped by stage of diabetic retinopathy

Stage of DR (number of eyes)	Mild DR (29)	Moderate DR (15)	Severe DR (8)	Proliferative DR (31)	*p* Value
Visual acuity (LogMAR; mean ± SD)	0.23 ± 0.24	0.2 ±0.22	0.19 ±0.2	0.2 ±0.25	0.88
Central subfield thickness at first visit, μm (mean ±SD)	366.3 ± 87.8	401.0 ±120.0	428.4 ±103.8	376.5 ±123.7	0.96
Central subfield thickness at time of enrollment, μm (mean ±SD)	336.6 ±91.31	331.8 ±38.6	336.5 ±58.9	311.9 ±68.8	0.57
Number of IVIs (in the last year), mean ± SD	5.7 ±3.7	7.9 ±2.3	5.4 ±2.1	4.2 ±3.5	0.003
Anti-VEGF IVI, n (%)	25 (86.2)	15 (100)	6 (75)	27 (87.1)	0.341
Steroid IVI, n (%)	4 (13.8)	0 (0)	2 (25)	4 (12.9)	0.121

*DR* diabetic retinopathy, *LogMAR* logarithm of the minimal angle of resolution, *SD* standard deviation, *IVI* intravitreal injection, anti-*VEGF* anti-vascular endothelial growth factor antibody

### Periodontitis

**Fig. 1:**
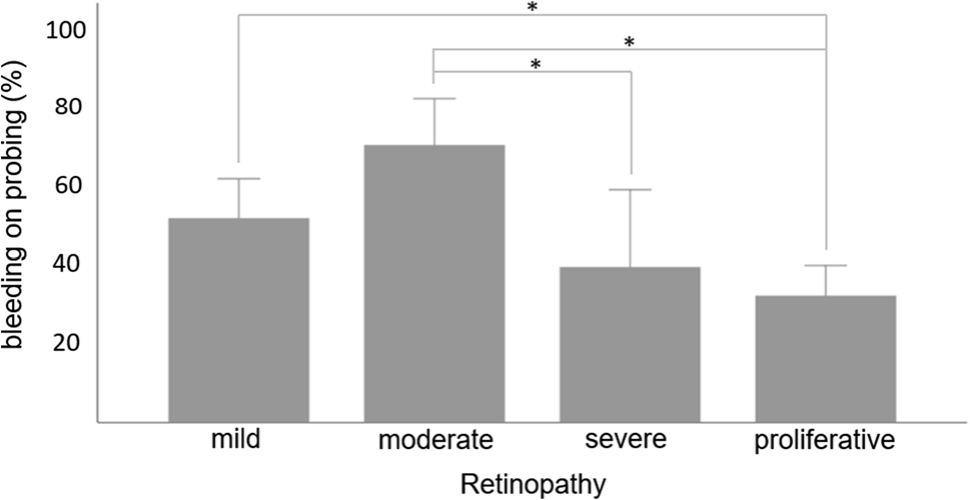
Bleeding on probing grouped by stage of diabetic retinopathy. The percentages of teeth that showed bleeding on probing grouped by the stage of diabetic retinopathy are presented. The asterisk brackets indicate significant differences between groups. The level of significance after adjustment for multiple testing is 0.0083

Five patients showed no signs of PD, eight (17.8%) had stage 1, 10 (22.2%) had stage 2, 17 (37.8%) had stage 3, and five (11.1%) had stage 4. Fourteen patients with mild DR (82.4%), 7 patients with moderate DR (87.5%), 4 patients with severe DR (100.0%), and 15 patients with proliferative DR (93.8%) had some degree of PD. Of the patients with some degree of PD, eight (17.8%) had grade A, 18 (40.0%) had grade B, and 14 (31.1%) had grade C. Patients with mild or moderate DR were more likely to have more severe periodontal conditions than patients with severe or proliferative DR (Table 3). Since the differences in plaque indices between type 1 DM and type 2 DM patients were significant (45.54 ± 11.2 vs. 67.5 ± 17.3, *p* = 0.001) and given the distinct nature of the two diseases, we split the groups for further analyses. BOP (Fig. 1) was significantly higher in patients with early stages of DR than in patients with late stages. Patients with a PISA > 500 mm^2^ (n = 10) needed significantly more IVIs than patients with a PISA < 500 mm^2^ (n = 25; 6.9 ± 3.1 versus 5.0 ± 3.5, *p* = 0.03). Overall, patients with high PISAs were more likely to have milder forms of DR and required more IVIs in the last year.

**Table 3:** Patient dental parameters grouped by stage of diabetic retinopathy

Type 1 diabetes					
Stage of DR (Number of eyes)	Mild DR (3)	Moderate DR (2)	Severe DR (2)	Proliferative DR (6)	*p* Value
Number of teeth	23 ± 1	23 ± 0	28 ± 0	26 ± 3	0.09
PI (%)	61 ± 17	43 ± 0	43 ± 0	40 ± 3	0.03
BOP (%)	81 ± 15	91 ± 0	30 ± 0	17 ± 6	0.001*
PPD ≥ 4 mm (n)	14.0 ± 5.2	19.0 ± 0.0	5.0 ± 0.0	4.0 ± 0.9	< 0.001*
CAL ≥ 4 mm (n)	15.1 ± 4.0	21.0 ± 0.0	5.0 ± 0.0	5.0 ± 0.9	< 0.001*
PISA, mm^2^ (mean ± SD)	624.2 ± 365.3	901.4 ± 0	331.6 ± 0	141.7 ± 74.8	0.004*
Type 2 diabetes					
Stage of DR (number of eyes)	Mild DR (26)	Moderate DR (13)	Severe DR (6)	Proliferative DR (25)	*p*-Value
Number of teeth	20 ± 5	23 ± 4	27 ± 4	23 ± 5	0.02
PI (%)	63 ± 19	70 ± 16	71 ± 3	70 ± 18	0.52
BOP (%)	51 ± 27	71 ± 23	45 ± 28	38 ± 22	0.001*
PPD ≥ 4 mm (n)	8.6 ± 5.2	16.5 ± 10.8	9.0 ± 7.2	8.2 ± 7.3	0.01
CAL ≥ 4 mm (n)	10.3 ± 5.4	16.5 ± 6.4	10.0 ± 7.6	11.2 ± 5.9	0.02
PISA, mm^2^ (mean ± SD)	366.7 ± 156.8	528.9 ± 283.2	310.6 ± 219.8	333.1 ± 176.3	0.03

The patients were split into type 1 diabetes and type 2 diabetes group to compare the periodontal parameters. The values reported for PPD and CAL ≥ 4 mm represent the number of teeth that meet these thresholds. *p Values that remained significant after adjustment for multiple testing (adjusted significance level is 0.0083)

*DR* diabetic retinopathy, *PI* plaque index, *BOP* bleeding on probing, *PISA* periodontal inflamed surface area, *PPD* probing pocket depth, *CAL* clinical attachment level

## Discussion

We evaluated PD in patients treated for DME. Patients with DME had a greater degree of periodontal inflammation when they were in the early stages of DR than when they were in the late stages. Furthermore, patients with severe periodontal inflammation (PISA > 500 mm^2^) needed significantly more IVIs per year than patients with low periodontal inflammation.

Other studies investigating the relationship between DR and PD showed that patients with a higher grade of PD had also a higher grade of DR. The association between PD and DR has been known for many years, with a study from 1988 reporting an increased percentage of DR in patients with PD [25]. Amiri et al. [26] found that patients with retinopathy had a greater community periodontal index of treatment needs (CPITN) than controls. A definite association between PD and the occurrence of DR was also described by Banthia et al. [27], which is in line with an investigation by Veena et al. showing an association between the severity of PD and DR [28]. A study by Sadzeviciene et al. [29] demonstrated that microvascular complications (including DR) were more frequently diagnosed in the presence of periodontal tissues showing a more severe inflammatory pathology. A survey of approximately 5000 patients with DM found an association between PD and DR and concluded that patients with DR should be referred for PD management [30]. Another study found that DR was associated with alveolar bone loss but not with PPD [31], highlighting the necessity of investigating various markers of PD simultaneously.

There are several differences between the previous studies and our study. The populations are of different ethnic origin. Our Austrian participants are older and have lower HbA1c levels and a longer duration of DM than the participants in the Iranian [26] and Indian study [28]. While only 40% of participants reported to have PD in a large Japanese survey [30], we found some degree of PD in almost 90% of our patients. Our study is the first to describe ophthalmological findings like retinal thickness and presence of DME.

The most important feature of our trial is that we did not only look for DR but for DME as well. While other studies do not describe the presence or absence of DME, all of our participants had DME. In this distinct subgroup of DR patients, we did not find the previously described correlation of DR and PD. In fact, patients with mild and moderate DR had more PD than patients with severe and proliferative DR. DME is driven by intraocular cytokine levels. We think that periodontal disease is more frequent in mild and moderate DR with DME, since subtle changes in the intraocular cytokine levels triggered by a remote inflammation like periodontal disease are relevant for the development of DME only in early stages of DR, while in later stages, intraocular cytokine levels are too high to be significantly influenced. While DR and PD may exhibit a bidirectional relationship during the years-long course of DM, DME, which can occur at any stage of DR, is more sensitive to factors inducing systemic inflammation levels. Our findings suggest that PD contributes to the pathogenesis of DME in the early stages of DR, when the slow but long-lasting local damage produced by DM has not yet outpaced the systemic inflammation driven by PD. A sizeable body of evidence supports our conclusions. Elevated levels of VEGF, interleukin (IL)-6, and IL-8 have been found in the aqueous humor of patients with DME [32], and systemic IL-6 levels have been found to be predictors of DME [33]. Noma et al. [14] found that IL-6 levels in the vitreous correlated with the severity of PD. Elevated levels of cytokines in the vitreous are related to retinal vascular permeability and the severity of DME [34]. This evidence suggests the biological plausibility of an association between PD and macular edema.

We found that patients with lower PISA needed less IVIs. Since PISA reflects the inflammatory burden of periodontitis and predicts HbA1c levels most accurately [35], we used PISA to further determine associations with the number of IVIs needed. The role of inflammatory pathways in diabetes and diabetes-associated complications is emerging, and there is an increasing interest in targeting inflammation for disease prevention and control. Interestingly, systemic anti-inflammatory treatments have been studied in clinical trials for DME, including nonsteroidal anti-inflammatory drugs (NSAIDs) [36], infliximab (TNF-α antibody) [37], and canakinumab (IL-1β antibody) [38], which have shown promising effects. It is well established that the treatment of PD leads to a significant reduction in systemic inflammatory marker levels [39]. Together with our findings that patients with high PISA values needed significantly more IVIs to treat DME, these data suggest that PD should be considered a therapeutic target for treating DM and DME. On average, patients with PISAs > 500 mm^2^ received approximately 7 IVIs per year, whereas patients with PISAs < 500 mm^2^ received approximately 5 IVIs per year. This result aligns with other studies showing associations of other complications of DM, such as diabetic kidney disease, with the number of IVIs needed per year [40]. Therefore, we recommend treating PD in patients with DME, especially in the early course of DM. Whether PD treatment could lead to a reduced frequency of IVIs needs to be investigated in future studies.

There are several limitations to our study. We did not measure cytokine levels in the vitreous or aqueous humor, which could have further improved the assessment of inflammation. Finally, we had resources for only a cross-sectional study design. Future longitudinal studies will investigate the benefits of PD treatment for DME.

In conclusion, we found more bleeding on probing and higher PISA values in patients treated for DME with early stages of DR than in patients with late stages of DR. Moreover, patients with higher PISA values also received significantly more IVIs for DME treatment. Further prospective studies are necessary to confirm the association of DME and PD and the possible impact of periodontal treatment on DME.
